# MOLECULAR SENESCENCE IS ASSOCIATED WITH DEMENTIA RISK IN THE ATHEROSCLEROSIS RISK IN COMMUNITIES STUDY

**DOI:** 10.1093/geroni/igae098.2297

**Published:** 2024-12-31

**Authors:** Anna Prizment, Saeun Park, Sanaz Sedaghat, Shuo Wang, Weihua Guan, Weihong Tang, Keenan Walker, Timothy Hughes

**Affiliations:** University of Minnesota, Minneapolis, Minnesota, United States; University of Minnesota, Minneapolis, Minnesota, United States; University of Minnesota, Minneapolis, Minnesota, United States; University of Minnesota, Minneapolis, Minnesota, United States; University of Minnesota, Minneapolis, Minnesota, United States; University of Minnesota, Minneapolis, Minnesota, United States; National Institute on Aging, Baltimore, Maryland, United States; Wake Forest University School of Medicine, Winston-Salem, North Carolina, United States

## Abstract

Recent evidence suggests that the accumulation of senescent cells induces chronic inflammation via the senescence-associated secretory phenotype (SASP), increasing Alzheimer’s disease risk. However, the association between SASP proteins and dementia risk remains unknown. Our objective was to develop SASP scores using various protein panels and evaluate their relationship with dementia risk in Atherosclerosis Risk in Communities (ARIC) study – a prospective cohort of 11,755 White and Black men and women (46-70 years old) at baseline (1990-92). We created 3 SASP scores by training them against incident dementia (1,493 cases) in 7,837 participants (67%) followed until 2019. The first score (Score-1), created by Ridge regression, included 133 proteins identified in Basisty’s atlas (2020). The second score (Score-2) included 16 dementia-associated proteins (of 133 SASP proteins). The third score (Score-3) was based on 7 SASP proteins validated by Schafer (2020); it included only 2 dementia-associated proteins: growth differentiation factor 15 (GDF15) and activin A. These scores were computed as weighted sums of standardized SASP proteins (mean=0, SD=1) in the test set of the remaining 3,918 participants (758 dementia cases). Multivariable Cox regression estimated the associations between each score and incident dementia. Hazard ratio (HR, 95% CI) per SD were for Score-1 (1.33, 1.23-1.45), Score-2 (1.20, 1.11-1.31), and Score-3 (1.23, 1.13-1.34), all p< 0.001. Thus, the 3 SASP scores, consisting of different protein numbers, showed comparable positive associations with dementia risk. SASP scores, even those consisting of a small number of proteins, may serve as a potential biomarker for dementia.

